# The inverse relationship between solar-induced fluorescence yield and photosynthetic capacity: benefits for field phenotyping

**DOI:** 10.1093/jxb/eraa537

**Published:** 2020-12-19

**Authors:** Peng Fu, Katherine Meacham-Hensold, Matthew H Siebers, Carl J Bernacchi

**Affiliations:** 1 Carl R. Woese Institute for Genomic Biology, University of Illinois at Urbana-Champaign, Urbana, IL, USA; 2 Department of Plant Biology, University of Illinois at Urbana-Champaign, Urbana, IL, USA; 3 USDA-ARS Global Change and Photosynthesis Research Unit, Urbana, IL, USA; 4 Bielefeld University, Germany

**Keywords:** Gas exchange, hyperspectral images, phenotyping, photosynthesis, plant breeding, solar-induced fluorescence

## Abstract

Improving photosynthesis is considered a promising way to increase crop yield to feed a growing population. Realizing this goal requires non-destructive techniques to quantify photosynthetic variation among crop cultivars. Despite existing remote sensing-based approaches, it remains a question whether solar-induced fluorescence (SIF) can facilitate screening crop cultivars of improved photosynthetic capacity in plant breeding trials. Here we tested a hypothesis that SIF yield rather than SIF had a better relationship with the maximum electron transport rate (*J*_max_). Time-synchronized hyperspectral images and irradiance spectra of sunlight under clear-sky conditions were combined to estimate SIF and SIF yield, which were then correlated with ground-truth *V*_cmax_ and *J*_max_. With observations binned over time (i.e. group 1: 6, 7, and 12 July 2017; group 2: 31 July and 18 August 2017; and group 3: 24 and 25 July 2018), SIF yield showed a stronger negative relationship, compared with SIF, with photosynthetic variables. Using SIF yield for *J*_max_ (*V*_cmax_) predictions, the regression analysis exhibited an *R*^2^ of 0.62 (0.71) and root mean square error (RMSE) of 11.88 (46.86) μmol m^–2^ s^–1^ for group 1, an *R*^2^ of 0.85 (0.72) and RMSE of 13.51 (49.32) μmol m^–2^ s^–1^ for group 2, and an *R*^2^ of 0.92 (0.87) and RMSE of 15.23 (30.29) μmol m^–2^ s^–1^ for group 3. The combined use of hyperspectral images and irradiance measurements provides an alternative yet promising approach to characterization of photosynthetic parameters at plot level.

## Teaser

A new approach to predict photosynthetic capacity based on solar-induced fluorescence yield is presented, which offers high-throughput screening of cultivars for improved photosynthesis.

## Introduction

Improving photosynthesis is regarded as a promising means by which crop yields can be improved to meet increasing pressure on global agricultural yields (Parry *et al.*, 2010; Long *et al.*, 2015; Ort *et al.*, 2015). Research efforts are underway to explore photosynthetic variation among both genetically modified and wild-type crop cultivars (von Caemmerer and Evans, 2010; Lawson *et al.*, 2012; Evans, 2013; Andralojc *et al.*, 2018) for which accurate and rapid measurements of photosynthetic capacity are required. For C_3_ crops, photosynthetic capacity is primarily determined by the maximum rate of Rubisco carboxylation (*V*_cmax_) and the maximum electron transport rate (*J*_max_) (Farquhar *et al.*, 1980; Bernacchi *et al.*, 2003). Traditionally, *V*_cmax_ and *J*_max_ are measured using leaf gas exchange which is both time-consuming and labor-intensive, and thus an impractical solution to quantify photosynthetic variation for hundreds and thousands of crop cultivars investigated in a crop breeding context (Tester and Langridge, 2010; Furbank and Tester, 2011; Araus and Cairns, 2014). Thus, advanced techniques to characterize variations of photosynthetic capacity accurately in a rapid manner at field scale are greatly needed to accelerate selection of crop cultivars with improved photosynthesis (Furbank *et al.*, 2019).

Numerous methods have been developed to estimate photosynthetic capacity spatially and temporally using remotely sensed data, primarily for improved mapping and modeling of gross primary productivity (GPP) at regional and global scales (e.g. Sims *et al.*, 2008, Houborg *et al.*, 2013, Serbin *et al.*, 2015), and He *et al.*, 2019). These methods may also be applied at field scale to assess photosynthetic performance of crop cultivars in breeding trials using high-throughput phenotyping platforms (HTPPs) mounted with (hyper)spectral sensors (Camino *et al.*, 2019; Fu *et al.*, 2019; Meacham-Hensold *et al.*, 2019). Remote sensing techniques to estimate photosynthetic capacity generally fall into three categories depending on proxy variables [i.e. reflectance, leaf traits, and solar-induced fluorescence (SIF)] used to build the empirical relationship.

The first category involves directly relating reflectance spectra in a few of absorption/reflection wavelengths (e.g. spectral indices) or full spectra (400–2500 nm) to photosynthetic variables (i.e. *V*_cmax_ and *J*_max_) using machine learning algorithms (Serbin *et al.*, 2012; Heckmann *et al.*, 2017; Yendrek *et al.*, 2017; Meacham-Hensold *et al.*, 2019; Fu *et al.*, 2020). However, understanding of the underlying mechanisms for predicting photosynthetic capacity from reflectance spectra and indices remains largely unsolved. This lack of explainable features limits extrapolation of predictions of photosynthetic variation to other species or crop cultivars under various environmental conditions (Fu *et al.*, 2019).

The second category uses remotely estimated plant functional traits such as leaf nitrogen as proxies for photosynthetic capacity (Kattge *et al.*, 2009; Walker *et al.*, 2014). These plant function traits are generally correlated with enzymes or light-harvesting pigments that can modulate the photosynthesis process. For example, the photosynthetic carbon-assimilating enzyme Rubisco is the dominant protein found in leaf material and thus is highly correlated with nitrogen concentration (Evans, 1989). More recently, studies have suggested that leaf chlorophyll, responsible for light harvesting in photosynthesis, would be a better proxy than leaf nitrogen for seasonal variations in photosynthetic capacity (Houborg *et al.*, 2013; Alton, 2017; Croft *et al.*, 2017). Despite success in mapping seasonal variations in photosynthetic capacity, this group of techniques may not be directly applicable at field scale in a plant breeding context since spurious variations in reflectance spectra incurred by plant geometry and soil background are not easily accounted for with HTPP to estimate leaf chlorophyll or nitrogen (Jay *et al.*, 2016; Mohd Asaari *et al.*, 2018). Thus, estimations of photosynthetic capacity suffer the error propagation from empirical or machine learning models used to retrieve leaf pigments. Additionally, recent work shows that the relationship between photosynthetic capacity and leaf nitrogen may not hold for genetically modified crop cultivars (Meacham-Hensold *et al.*, 2019). Leaf chlorophyll was also shown to exhibit a worse relationship with photosynthetic variables (*R*^2^ of <<0.5) compared with reflectance spectra (*R*^2^ of ~0.8) at small field scale (Fu *et al.*, 2020).

The third category of techniques is based on SIF as an indicator for photosynthetic activity (Zhang *et al.*, 2014, 2018). SIF represents light emission resulting from excited chlorophyll molecules and competes with photochemistry and non-photochemical quenching (NPQ) pathways for de-excitation (Porcar-Castell *et al.*, 2014). Thus, changes in SIF can be used to probe the photosynthetic apparatus and CO_2_ exchange at various spatial scales. SIF has been shown to have a quasi-linear relationship with canopy-scale photosynthesis, generally referred to as GPP, for various ecosystems (Frankenberg *et al.*, 2011; Yang *et al.*, 2017; Li *et al.*, 2018), and therefore is not generally associated with the underlying photosynthetic physiology. Evidence suggests that the relationship between GPP and SIF becomes more linear with increasing spatial and temporal extents (Guanter *et al.*, 2012; Sun *et al.*, 2017; Verma *et al.*, 2017; Miao *et al.*, 2018; Yang *et al.*, 2018; Magney *et al.*, 2019). Thus, SIF as a proxy of photosynthesis for small-field plots may be problematic (e.g. 1 m^2^ in plant breeding trials) since only a few selected dates of gas exchange measurements associated with photosynthetic variations are collected (Fu *et al.*, 2019; Meacham-Hensold *et al.*, 2019). Recent analysis using the SCOPE (Soil Canopy Observation, Photochemistry and Energy Fluxes) model shows that SIF is more related to canopy properties such as chlorophyll content, leaf area index, and leaf angle distribution than *V*_cmax_ (Koffi *et al.*, 2015; Verrelst *et al.*, 2015). As such, further studies are warranted to evaluate the feasibility of SIF to estimate photosynthetic capacity particularly at the field scale that may provide mechanistic linkages between SIF and photosynthetic physiology.

As HTPPs with spectroradiometers and cameras of high spectral resolution are widely used for plant phenotyping (e.g. Araus *et al.*, 2018), there exist opportunities to evaluate SIF-based methods for identifying differences in photosynthetic capacities among crop cultivars at plot level based on millimeter spatial resolution hyperspectral imagery. At present, it remains uncertain whether SIF-associated signals can be used as proxies for photosynthetic capacity including *V*_cmax_ and *J*_max_ at plot scales in a high-throughput phenotyping context. As light absorbed by plants can experience one of three fates (photochemistry, NPQ, or SIF) in competition, the increase in yield of one will result in a decrease in yield of the other two (Maxwell and Johnson, 2000; Müller *et al.*, 2001). Thus, we hypothesize that the increase of SIF yield [defined as the ratio between SIF and absorbed photosynthetically active radiation (PAR)] would lead to a decrease of the electron transport rate (i.e. *J*_max_) under saturated light conditions. Given the competing relationship of electrons for three competitive fates, it follows that electron transport capacity (*J*_max_) decreases as SIF yield increases. Thus, *J*_max_ was used to test the hypothesis. The hypothesis is only made to *J*_max_ since SIF can vary as a result of availability of electron acceptors in the PSII, and NPQ measurements are not obtainable with the current phenotyping platform and sensors. Furthermore, because PSII electron transport is correlated well with CO_2_ fixation (i.e. the correlation between *V*_cmax_ and *J*_max_) (Edwards and Baker, 1993), we hypothesize that the SIF yield will also have a close relationship to *V*_cmax_ but not as strong as the relationship to *J*_max_. This weaker relationship is predicted since fluorescence capture is more closely linked with the electron transport chain (represented as *J*_max_) while rates of CO_2_ fixation may compete with other processes such as photorespiration, nitrogen metabolism, and electron donation to oxygen (the Mehler reaction).

By addressing these hypotheses, this study differs from previous studies in using satellite-based SIF measurement to probe photosynthesis (or GPP at the ecosystem level) by providing a more in-depth investigation of the possible mechanistic linkages between SIF-related information and photosynthetic physiology. The objective of this study is to explore means, through the combined use of sensors, for high-throughput screening of crop trials aimed at selecting cultivars for improved photosynthetic performance at the plot level.

## Materials and methods

### Plant materials and experimental design

Both wild-type and genetically modified tobacco (*Nicotiana tabacum*) cultivars (totaling 10 cultivars) were used to evaluate SIF/SIF yield as a proxy for photosynthetic capacity. These cultivars exhibited large variations in photosynthetic traits ranging from 15.98 μmol m^–2^ s^–1^ to 318.96 μmol m^–2^ s^–1^ for *V*_cma*x*_, and from 118.85 μmol m^–2^ s^–1^ to 338.70 μmol m^–2^ s^–1^ for *J*_max_ (Fu *et al.*, 2020). The genetically modified lines have alterations to the photosynthetic pathway including increased carbon reduction enzymes, a photorespiratory bypass, and lines with increased electron transport in metabolite pools, leading to *V*_cmax_ or *J*_max_ >300.00 μmol m^–2^ s^–1^. Further descriptions of these cultivars can be found in Meacham-Hensold *et al.* (2019, 2020).

Cultivar seedlings were germinated in a greenhouse and then transplanted to the field site of the University of Illinois Energy Farm (40.063°N, 88.207°W; descriptions of this farm are available at http://energyfarm.illinois.edu/index.html) at the four-leaf stage. Two weeks before transplanting, the field site was fertilized with a high level of nitrogen (ESN Smart Nitrogen, 310 kg ha^–1^, ~150 ppm). In addition, the field site was controlled for tobacco pests using a biological pesticide *Bacillus thuringiensis* v. *kurstaki* (54%) (DiPel PRO) applied first at 5 d before transplanting and then at bi-weekly intervals. Two days before transplanting, a broad action herbicide, glyphosate-isopropylammonium (41%) (Killzall; VPG) (15 liters at 70 g l^–1^) was also applied to the field site. Throughout plant growth, irrigation was provided as needed. Each tobacco cultivar was planted in four replicated plots arranged in a 6×6 grid (36 plants per plot) with 0.38 m spacing between plantings. Field measurements associated with hyperspectral reflectance, gas exchange, and irradiance/radiance were made on various dates—6, 7, 12, and 31 July and 18 August 2017, and 24 and 25 July 2018—under clear-sky conditions.

### Collection of hyperspectral images and irradiance measurements

**Fig. 1.**
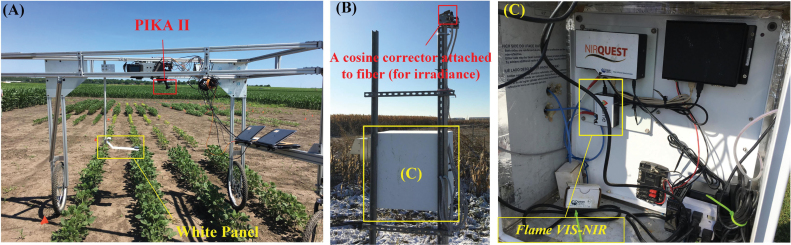
The phenotyping platform (A) mounted with the PIKA II camera (400–900 nm) and a temperature-controlled enclosure (B) with the Flame VIS-NIR spectrometer (C) installed. The bare optic fiber of the Flame spectrometer was attached to a cosine corrector, facing toward the sky to record irradiance spectra of sunlight.

The Resonon PIKA II VNIR hyperspectral imaging camera (Resonon Inc., Bozeman, MT, USA) mounted on a phenotyping platform (Fig. 1A) was used to collect hyperspectral images for each plot. The camera used a push-broom design to scan each tobacco plot with 640 spatial channels at a height of 1.6 m from the ground. Image acquisition was controlled using the SpectrononPro software (Resonon Inc.) and completed within the time window between 10.00 h and 14.30 h local time under clear-sky conditions. Collected images had a spectral resolution of 2.1 nm (240 spectral bands in total from 400 nm to 900 nm, with a signal to noise ratio of ~300) and a spatial resolution of 0.1 mm at nadir. For reflectance conversion, a 99% reflective white panel (Labsphere Inc., North Dutton, NH, USA) was mounted horizontally above the top of the plant canopy and in the field of view of the camera (Fig. 1A). Prior to each image scan, camera integration time was carefully set to ~15% below saturation using the radiance signal from the white panel. The hyperspectral camera was calibrated spectrally (the camera’s sensitivity to light intensity), radiometrically (conversion of image digital numbers to radiance), and spatially (consistent spectral response curves among different spatial channels) prior to collection of plot images.

The downwelling irradiance measurements were made using a spectrometer (Flame VIS-NIR, Ocean Optics Inc., Largo, FL, USA) that was calibrated radiometrically and spectrally with a standard light source (HL-3p-CAL, Ocean Optics Inc.). The spectrometer covered the spectral region from 350 nm to 1000 nm with a spectral resolution of ~0.35 nm. A cosine corrector was attached to the fiber of the spectrometer to have a field view of 180°, facing toward the sky to record the irradiance spectra of the sunlight. The spectrometer and its associated accessories and miniaturized computers were installed in a temperature-controlled enclosure (Fig. 1B) at the edge of the tobacco plots. For each measurement day, the irradiance spectra were acquired at a frequency varying from 20 Hz to 50 Hz (depending on the sunlit intensity) throughout the time period of hyperspectral image collections. Hyperspectral images and irradiance spectra were matched based on time stamps for calculating SIF and SIF yield. The irradiance measurements (~0.35 nm spectral resolution) were resampled to 2.1 nm spectral resolution using the spline interpolation method.

### Gas exchange measurements

Photosynthetic capacities, *V*_cmax_ and *J*_max_, were determined from response curves of photosynthesis (*A*) to a series of intercellular CO_2_ concentrations (*C*_i_), namely 400, 200, 50, 100, 300, 400, 600, 900, 1200, 1500, 1800, and 2000 μmol mol^–1^, using a mathematical model (Farquhar *et al.*, 1980; Bernacchi *et al.*, 2003; Sharkey *et al.*, 2007). These *A*/*C*_i_ response curves were recorded by a portable infrared gas analyzer (LI-6800, LICOR Biosciences, Lincoln, NE, USA) within 30 min of image acquisition on three sunlit, last fully expanded leaves per plot. Prior to the CO_2_ response analysis, light response curves were carried out on all genotypes used in this study to determine the light saturation point. Subsequently PAR inside the chamber head was set at 1800 μmol m^–2^ s^–1^ to ensure saturating light conditions for each CO_2_ response curve, allowing confidence in true *J*_max_ under the given conditions.

Leaf temperatures of these three leaves were measured with an FLR TG54 handheld IR gun, and the air temperature of the gas exchange cuvette was set to the mean of the three temperature values. Inside the gas exchange chamber, relatively humidity was controlled to 65%. Prior to each *A/C*_i_ curve, leaves were acclimated to chamber conditions for a minimum of 160 s. The minimum and maximum wait time before each individual measurement of a response curve was 160 s and 200 s, respectively. Mesophyll conductance (*g*_m_) was calculated using the linear dependence of *g*_m_ on temperature with known values for tobacco at 25 °C reported in von Caemmerer and Evans (2015). Finally, a total of 108 and 81 leaf level values of *V*_cmax_ and *J*_max_, respectively, were collected. *V*_cmax_ and *J*_max_ values were then averaged from three leaves per plot for 36 and 27 total plots, respectively. One of the genetically modified tobacco cultivars, double Rubisco knockdown plants (SSuD) used in this study, was not electron transport limited under any conditions (Meacham-Hensold *et al.*, 2019), leading to fewer measurements of *J*_max_ than of *V*_cmax_.

### Analysis techniques

**Fig. 2.**
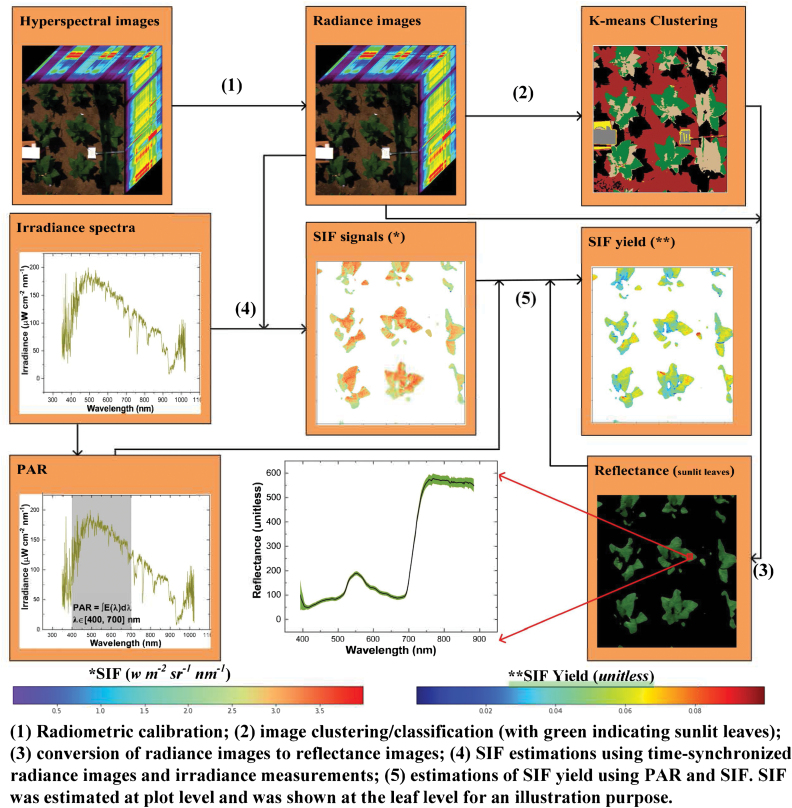
The data analysis flowchart for retrieving SIF and SIF yield using time-synchronized hyperspectral images and irradiance spectra for each selected clear-sky day of measurements. SIF, solar-induced fluorescence; PAR, photosynthetically active radiation (400–700 nm).

Figure 2 outlines the five steps (i.e. 1–5) for retrieving SIF and SIF yield on a per-pixel basis. These data analysis steps were based on time-synchronized hyperspectral images and irradiance spectra collected in selected clear-sky days of measurements. The first part of the data analysis (steps 1–3) consisted of radiometric calibration of raw images to radiance images and then the classification of radiance images for deriving reflectance images. The second part included estimations of SIF data using downwelling irradiance spectra and hyperspectral images, and estimations of SIF yield using SIF data and PAR (400–700 nm), for each plot. These SIF and SIF yield data at plot level were then correlated with *V*_cmax_ and *J*_ma*x*_ to evaluate whether SIF/SIF yield would be a good proxy for photosynthetic capacities.

### Image processing

The conversion of raw hyperspectral images (digital numbers with a 12-bit depth) to reflectance images of sunlit leaves was implemented in a customized programming system coded in Python (Python Software Foundation, https://www.python.org/) developed by Fu *et al.* (2020). First, digital numbers from raw images were converted to absolute spectral radiance (unit: w m^–2^ sr^–1^ nm^–1^) using calibration files provided by the instrument company. Second, an unsupervised classification algorithm—the *k*-means clustering algorithm—was applied to radiance images for classification. In the clustering algorithm, the number of clusters was set at six. Among these clusters, the one with the highest mean radiance value was identified as a white panel. As the reflectance spectrum of the white panel was known, reflectance images of all clusters were calculated using Equation 1.

$$\begin{array}{l} R=\frac{S_{clusters}}{S_{white}}*R_{white} \end{array}$$ (1)

where *S*_clusters_ and *S*_white_ are radiance values from each cluster and the white panel, respectively, *R*_white_ refers to the reflectance of the white panel calibrated and provided by Labsphere, and *R* is the absolute reflectance of each cluster.

With a normalized difference vegetation index (NDVI) value (as shown in Equation 2; Tucker, 1979) >0.1, two clusters—sunlit leaves and leaves in shadow—were clearly delineated. The cluster of sunlit leaves was identified further as it had a higher mean spectral radiance than the other cluster.

$$\begin{array}{l} NDVI=\;\frac{R_{770-780\;nm\;}-\;R_{650-660\;nm}}{R_{770-780\;nm}\;+\;R_{650-660\;nm}} \end{array}$$ (2)

where *R*_770–780 nm_ and *R*_650–660 nm_ refer to mean reflectance values within 770–780 nm and 650–660 nm, respectively. Only spectra of sunlit leaves were used for SIF estimations and the regression analysis of SIF/SIF yield with photosynthetic capacities (*V*_cmax_ and *J*_max_).

### Retrieval of SIF and SIF yield

With the time-synchronized irradiance measurements and hyperspectral radiance images, SIF data were computed using the improved Fraunhofer line discrimination (iFLD) method (Alonso *et al.*, 2008). The iFLD method relies on two radiance measurements, one inside and one outside a Fraunhofer line (761 nm in this study, O_2_ A-band), and apparent correction factors, as shown in Equation 3.

$$\begin{array}{l} SIF=\;\frac{\alpha_{R}E\left(\lambda_{out}\right)*L\left(\lambda_{in}\right)-E\left(\lambda_{in}\right)*L\left(\lambda_{out}\right)}{\alpha_{R}E\left(\lambda_{out}\right)-\alpha_{F}E\left(\lambda_{in}\right)} \end{array}$$ (3)

where *E*(λ _out_) and *E*(λ _in_) refer to irradiance signals measured outside and inside the dark line (i.e. 761 nm) from the FLAME VIS-NIR spectrometer, and *L*(λ _in_) and *L*(λ _out_) are radiance signals measured inside and outside the dark line provided by the PIKA II hyperspectral camera. The two coefficients α _R_ and α _F_ are used to characterize variations in the fluorescence and the reflectance values inside and outside the absorption bands following Alonso *et al.* (2008). In this study, λ _in_ and λ _out_ were set to 761 nm and 754 nm, respectively, to facilitate estimation of SIF. Only SIF from the O_2_ A-band is estimated using Equation 3 since the iFLD method cannot accurately estimate SIF from the O_2_ B-band (or at least the estimation accuracy for the O_2_ B-band is not as good as that for the O_2_ A-band). According to Alonso *et al.* (2008), the SIF retrieval error for the O_2_ A-band is in the order of 10^–2^. Further details of the iFLD method can be found in Alonso *et al.* (2008). To partly remove the scan angle effects of the hyperspectral camera (scan angle of ±46.1°), only pixels within the angle view of ±15° (near-nadir and nadir view) were used. The solar zenith angle was <35° over the study period. Although the spectral fitting methods (e.g. Meroni and Colombo, 2006) have commonly been used to estimate SIF recently, in this study the spectral resolution (2.1 nm with a signal to noise ratio of ~200) of the PIKA II hyperspectral camera limits the use of such methods (since only six irradiance and radiance measurement pairs available around 761 nm are not enough for spectral fitting). The use of the iFLD method to estimate SIF has been shown in recent studies that have suggested reasonable SIF retrievals can be achieved using broader spectral bandwidth (i.e. ~2 nm spectral sampling interval, signal to noise ratio ~300) (Damm *et al.*, 2011; Camino *et al.*, 2019).

After the SIF calculation, the SIF yield was estimated following Equation 4.

$$\begin{array}{l} \left\{ \begin{array}{l} APAR=PAR*fAPAR\\ fAPAR=NDVI\\ PAR=\;\underset{400}{\overset{700}{\int }}\;E\left(\lambda \right)\;d\lambda \\ SIF_{y}=SIF/APAR \end{array}\right. \end{array}$$ (4)

where SIF_y_ refers to the SIF yield (also known as apparent SIF yield), PAR is photosynthetically active radiation integrated from 400 nm to 700 nm using the downwelling irradiance measurements, APAR is absorbed photosynthetically active radiation, fAPAR is the ratio between APAR and PAR. In this study, NDVI (Equation 2) was used as a proxy of fAPAR.

### Regression analysis

SIF/SIF yield values at plot level were correlated with the corresponding *V*_cmax_ and *J*_max_ using the linear regression analysis. The performance of the regression model to predict *V*_cmax_ and *J*_max_ was evaluated based on the coefficient of determination (*R*^2^) and RMSE. The SIF and SIF yield data were also grouped by their time proximity for regression analysis, namely 6, 7, and 12 July 2017 for group 1 (14 measurements for *V*_cmax_ and 11 for *J*_max_ at plot level), 31 July and 18 August 2017 for group 2 (12 measurements for *V*_cmax_ and 8 for *J*_max_ at plot level), and 24 and 25 July 2018 for group 3 (10 measurements for *V*_cmax_ and 8 for *J*_max_) at plot level). The Pearson’s correlation coefficient (CC) was also used to analyze the relationship between *V*_cmax_ and *J*_max_.

## Results

### SIF and SIF yield as proxies of *J*_max_

**Fig. 3.**
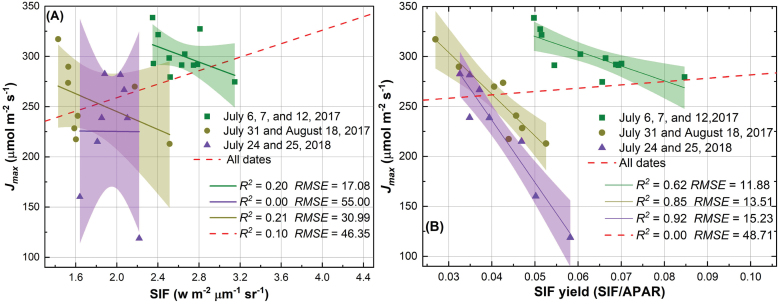
The relationship between SIF and *J*_max_ (A), and the relationship between SIF yield and *J*_max_ (B). The shaded area shows the 95% confidence interval of the regression line.

Both SIF and SIF yield were correlated with photosynthetic capacity *J*_max_ using regression analysis. When SIF data of all measurement days were considered for predicting *J*_max_, the regression performance was significant (*R*^2^=0.10, RMSE=46.35 μmol m^–2^ s^–1^; Fig. 3A). Using SIF yield of all measurement days, however, the regression analysis was not statistically significant (*P*-value >0.05; *R*^2^=0.00; Fig. 3B). When observations were binned by measurement days (i.e. group 1: 6, 7, and 12 July 2017; group 2: 31 July and 18 August 2017; and group 3: 24 and 25 July 2018), SIF yield exhibited a stronger relationship, compared with SIF, with photosynthetic capacity *J*_max_ (Fig. 3). More specifically, the regression of *J*_max_ using SIF yield showed an *R*^2^ of 0.62 and RMSE of 11.88 μmol m^–2^ s^–1^ for group 1, an *R*^2^ of 0.85 and RMSE of 13.51 μmol m^–2^ s^–1^ for group 2, and an *R*^2^ of 0.92 and RMSE of 15.23 μmol m^–2^ s^–1^ for group 3 (Fig. 3B). In contrast, regression of *J*_max_ using SIF generally showed a smaller *R*^2^ (<0.25) and greater RMSE (>17 μmol m^–2^ s^–1^). In addition, it was found that the relationship between SIF and *J*_max_ was not consistent over time as both negative and positive correlations were observed for binned groups (Fig. 3A). Figure 3B shows that the best performance of the regression model to predict *J*_max_ was achieved for group 3 (*R*^2^=0.92, RMSE=15.23 μmol m^–2^ s^–1^).

### SIF and SIF yield as proxies of *V*_cmax_

**Fig. 4.**
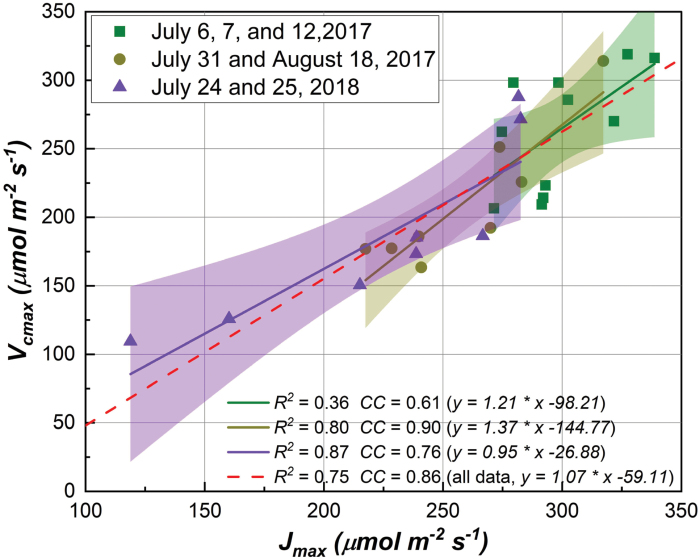
The relationship between *V*_cmax_ and *J*_max_ for each binned time group and among all time groups. The shaded area shows the 95% confidence interval of the regression line (i.e. using *J*_max_ to predict *V*_cmax_). CC refers to the Pearson’s correlation coefficient between *V*_cmax_ and *J*_max_.

**Fig. 5.**
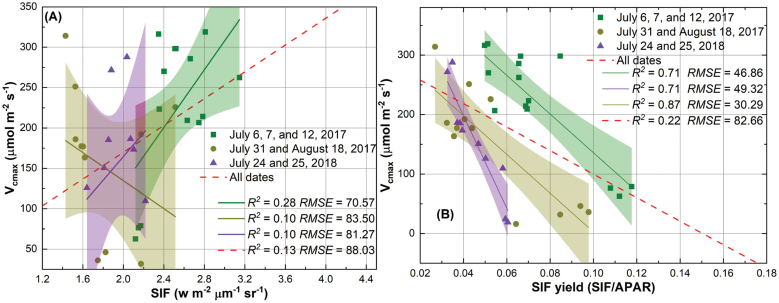
The relationship between SIF and *V*_cmax_ (A), and the relationship between SIF yield and *V*_cmax_ (B). The shaded area shows the 95% confidence interval of the regression line.

Figure 4 shows that *V*_cmax_ and *J*_max_ were highly correlated within each binned group and among all data groups. When measurements of all days were used, variations in *J*_max_ could explain 75% of variance in *V*_cmax_ (with an overall CC of 0.86; Fig. 4). When measurements were binned by groups, a statistically significant relationship between *J*_max_ and *V*_cmax_ was still observed, namely a CC of 0.61 for group 1, a CC of 0.90 for group 2, and a CC of 0.76 for group 3. In addition, the slope of the regression between *J*_max_ and *V*_cmax_ only exhibited a small variation ranging from 0.95 to 1.37 among data groups. This strong relationship between *J*_max_ and *V*_cmax_ allowed further correlation between SIF/SIF yield and *V*_cmax_, as shown in Fig. 5.

Similar regression results were observed for *V*_cmax_ as compared with *J*_max_. When SIF yields of all measurement days were considered, the regression analysis yielded an *R*^2^ of 0.22 and RMSE of 82.66 μmol m^–2^ s^–1^ for predicting *V*_cmax_ (Fig. 5). Using SIF of all measurement days, the prediction performance for *V*_cmax_ was relatively worse (*R*^2^=0.13, RMSE=88.03 μmol m^–2^ s^–1^; Fig. 5). When observations were binned as groups, SIF yield exhibited a stronger relationship, compared with SIF, with photosynthetic *V*_cmax_ (Fig. 5). More specifically, regression analysis of *V*_cmax_ yielded an *R*^2^ of 0.71 and RMSE of 46.86 μmol m^–2^ s^–1^ for group 1, an *R*^2^ of 0.72 and RMSE of 49.32 μmol m^–2^ s^–1^ for group 2, and an *R*^2^ of 0.87 and RMSE of 30.29 μmol m^–2^ s^–1^ for group 3 (Fig. 5B). Regression of *V*_cmax_ using SIF generally showed a smaller *R*^2^ (<0.3) and greater RMSE (>70 μmol m^–2^ s^–1^ for *V*_cmax_ predictions). In addition, it was found that the relationship between SIF and *V*_cmax_ was not consistent over time as both negative and positive correlations were observed for binned groups (Fig. 5A). Still, the best performance of the regression model to predict *V*_cmax_ was achieved for group 3 (Fig. 5B).

### Explanation for the observed negative relationship between SIF yield and photosynthetic capacities

**Fig. 6.**
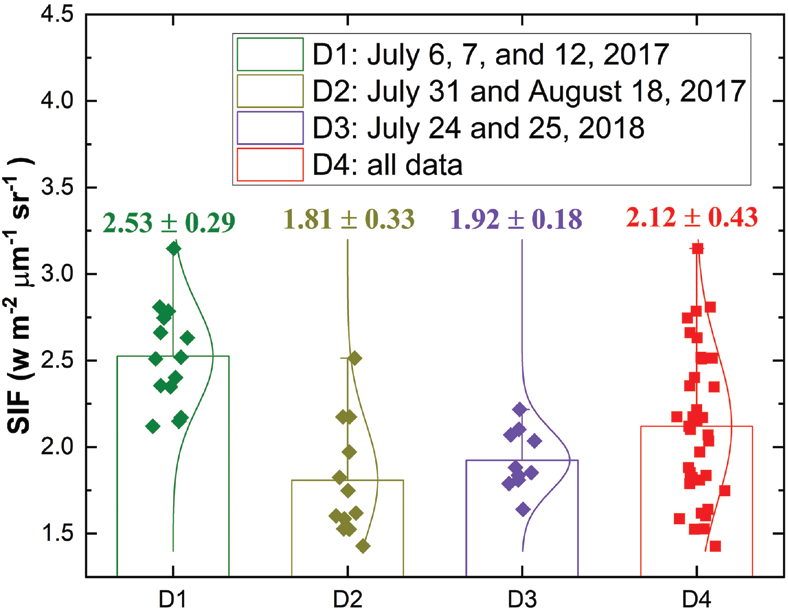
The statistical distribution of SIF within each of the three time periods (D1–D3) and among all three time periods (D4), 6, 7, and 12 July 2017, 31 July and 18 August 2017, and 24 and 25 July 2018. The numbers above each box show the mean and SD.

**Fig. 7.**
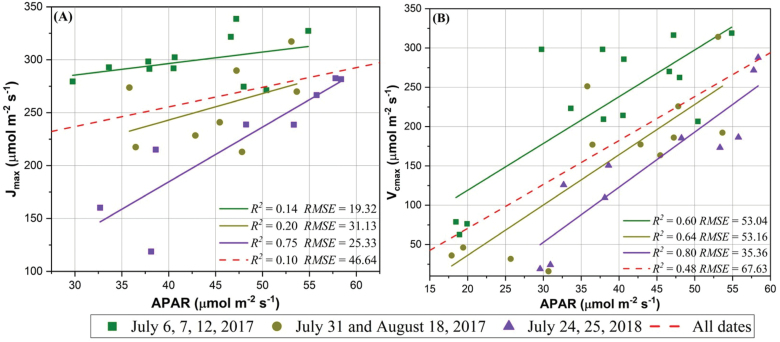
The relationship between APAR and (A) *J*_max_ and between APAR and (B) *V*_cmax_.

Analysis of all data combined, compared with those for binned groups, showed that SIF, in relative to SIF yield, has a relatively worse performance in estimating *V*_cmax_ or *J*_max_. This finding was reasonable as SIF data only exhibited a small variance among all three time periods and for each of the three time periods (Fig. 6). Within each time category (or data group), a strong observed negative relationship between photosynthetic capacity and SIF yield (Figs 3, 5) suggested that *V*_cmax_ and *J*_max_ were likely to be positively related to APAR (or NDVI×PAR), explaining the linkage between APAR and GPP at various spatial and temporal scales (Farquhar *et al.*, 1980; Viña and Gitelson, 2005; Miao *et al.*, 2018; Yang *et al.*, 2018). This positive relationship, as evidenced by Fig. 7, was consistent with that identified in previous studies relating photosynthetic capacities to various types of vegetation indices such as NDVI, structure insensitive pigment index, and ratio index (Zhang *et al.*, 2018; Fu *et al.*, 2020). Fu *et al.* (2020) showed that the NDVI-like index, if appropriately calculated with optimized combinations of spectral bands, could be used as a strong indicator (with a squared rank CC close to 0.8) for *V*_cmax_ and *J*_max_ using a similar dataset (with 11 tobacco cultivars rather than 10 cultivars) as in this study. Thus, the observed negative relationship between SIF yield and photosynthetic capacities was largely attributed to the well-known positive relationship between APAR (or NDVI×PAR) and photosynthetic capacities (based on Equation 4 in which APAR is the denominator). More specifically in this study, APAR (or NDVI×PAR) on average explained ~70% variation of *V*_cmax_ and ~35% variation of *J*_max_ (as supported by the average *R*^2^ values shown in Fig. 7A and B).

## Discussion

Although SIF is not a major pathway for de-excitation of chlorophyll, the results in Figs 3 and 5 suggested that variation in SIF yield (rather than SIF itself) could be used to examine the photosynthetic variable *J*_max_ and then *V*_cmax_ provided that there is a strong relationship between the two variables. The observed negative relationship in this study provided an alternative way to understand photosynthetic physiology at plot level, in addition to the well-known positive relationship between GPP and SIF reported in previous studies (Frankenberg *et al.*, 2011; Sun *et al.*, 2017; Yang *et al.*, 2017; Yang *et al.*, 2018). Other de-excitation pathways including NPQ that dissipates excess energy to heat through molecular vibrations, along with SIF, compete with the pathway through which photochemistry can occur at the reaction centers (Müller *et al.*, 2001). Even without quantification of NPQ, the results presented (Fig. 3) in this study supported our initial hypothesis that low photosynthetic capacity (i.e. *J*_max_) can lead to an increase in absorbed light energy to SIF for de-excitation for plants at saturated light. Here the SIF yield was a relative term (as normalized by the absorbed PAR), and the negative relationship did not contradict the previous finding of the positive relationship between GPP and SIF. It was also found that the relationship between SIF yield and *J*_max_ was generally stronger than that between SIF yield and *V*_cmax_ (except for group 1) when data were binned by groups. This is understandable given the close link between SIF and electron transport, and the challenges in quantifying Rubisco-dependent processes (*V*_cmax_) with a passive fluorescence detection (i.e. SIF). This finding corroborated our initial suggestion that SIF yield could also be used to understand the rates of CO_2_ fixation only if the correlation between *J*_max_ and *V*_cmax_ holds.

Compared with SIF or SIF yield, reflectance spectra (or hyperspectral data) have been more commonly used to estimate photosynthetic capacities in plant breeding programs to harness natural and/or artificially modified variations in photosynthesis for improved crop production (Serbin *et al.*, 2012; Silva-Perez *et al.*, 2017; Yendrek *et al.*, 2017; Fu *et al.*, 2019, 2020; Meacham-Hensold *et al.*, 2019). The studies using reflectance to estimate photosynthetic capacities may be attributable to the fact that reflectance is far easier to retrieve than SIF/SIF yield. Our previous study using a similar dataset suggested that the performance of hyperspectral data to estimate photosynthetic capacities was largely driven by measurements over different days (i.e. temporal variations in photosynthetic capacities; Fu *et al.*, 2020). As such, the prediction model cannot be built until the last day of measurements, greatly limiting the understanding of variations of photosynthesis among cultivars within the plant growth cycle. The identified relationship between SIF yield and photosynthetic capacities within each group of measurement days in Figs 3 and 5 provides an alternative yet promising mechanistic way to understand photosynthetic variations among wild and genetically modified cultivars. In particular, statistical models to predict photosynthetic capacities can be built with measurements over a short-term period, ranging from 2 d to 18 d, as shown in Figs 3 and 5. However, the optimal time interval used for binning measurements is not clear based on the current datasets, and further field measurements are needed to unravel whether environmental variables such as temperature and precipitation and phenological stages may also impact the binning interval. As the regression between SIF yield and *J*_max_ for all the three groups is statistically significant at a *P*-value <0.05, it is expected that the temporal interval for binning data may not greatly exceed 18 d, though further data pairs are needed to test this expectation.

The performance of SIF yield to predict photosynthetic capacities was similar to, if not better than, that of plant traits (such as leaf nitrogen and chlorophyll content) to estimate photosynthetic capacities (as shown in our own study; Fu *et al.*, 2020). Thus, the SIF yield as a proxy for photosynthetic capacities can be used as an alternative pre-screening technique in high-throughput phenotyping of crop trials. This technique is useful particularly when plant traits such as leaf nitrogen and chlorophyll content are decoupled from photosynthetic capacities through genetic engineering for improved photosynthesis (Long *et al.*, 2015; Ort *et al.*, 2015). In these cases, although plant traits may be retrieved from reflectance spectra at a high accuracy, the use of these plant traits such as leaf nitrogen and chlorophyll content as proxies for photosynthetic capacities could be problematic. This also makes the case for the radiative transfer models such as PROSEPCT (Jacquemoud and Baret, 1990) and SCOPE (van der Tol *et al.*, 2009) that are used for estimating plant traits and photosynthesis with inputs of reflectance spectra. The negative relationship between SIF yield and photosynthetic capacities presented in this study thus needs further examination to facilitate a better understanding between plant traits and photosynthesis in a more mechanistic way than possible using radiative transfer models.

The current study suggested that plant structure, associated with light capturing (i.e. APAR; e.g. Musavi *et al.*, 2016), played an important role in relating SIF yield to photosynthetic capacities, which is consistent with previous studies suggesting a better correlation of GPP with a structure parameter, NIRv, than SIF (Badgley *et al.*, 2017, 2019). The correlation of SIF yield with both *V*_cmax_ and *J*_max_ was attributed to the fact that *V*_cmax_ and *J*_max_ were tightly coupled (Wohlfahrt *et al.*, 1999; Kattge *et al.*, 2009). As a caveat, the presented approach may not work for crop cultivars and plants in which the correlation between *V*_cmax_ and *J*_max_ is likely to be shifted by leaf nitrogen, phosphorus, and specific leaf area (Walker *et al.*, 2014). Plus, recent efforts are being made to redesign photosynthetic process that may exhibit uncoupled correlations between *V*_cmax_ and *J*_max_ (Ort *et al.*, 2015).

Despite these promising results in using SIF yield as a proxy for photosynthetic capacities, the negative relationship shown in Figs 3 and 5 needs to be further examined with different cultivars and food crops such as maize and soybean. In this study, the calculation of SIF and SIF yield was simplified, for example, using NDVI as a proxy for the fAPAR, and the iFLD method for calculating SIF. For food crops, NDVI may suffer saturation at fAPAR beyond 0.7, and indices such as red-edge NDVI (Viña and Gitelson, 2005) and wide dynamic range vegetation index (Gitelson, 2004) may be better to approach fAPAR at high biomass density. However, this study showed a similar magnitude of SIF and SIF yield compared with previous studies (e.g. van der Tol *et al.*, 2014; Frankenberg and Berry, 2018).

The use of the iFLD method to estimate SIF can be refined in the future. Recent studies have suggested that reasonable SIF retrievals can be achieved using a broader spectral bandwidth, similar to the PIKA II camera with a ~2 nm spectral sampling interval and a signal to noise ratio of ~300 (Damm *et al.*, 2011; Camino *et al.*, 2019). Admittedly, biases in the SIF retrievals may arise from the broad spectral resolution; however, it is believed that consequences would be negligible for high-throughput phenotyping since the focus is on relative spatiotemporal variability rather than absolute SIF values (Camino *et al.*, 2019). In addition, SIF signals were obtained at a similar temperature over the study period (between 75 °F and 100 °F), removing impacts of temperature variations on the camera. According to the temperature experiment conducted within Resonon Inc. (data cannot be shared publicly), the shift in radiance caused by temperature effects is <0.11 w sr^–1^ μm^–1^ m^–2^ between 75 °F and 100 °F. This shift in radiance incurred by temperature is ~7% of SIF values (as SIF values were generally larger than 1.6 w sr^–1^ μm^–1^ m^–2^), suggesting that temperature impacts on SIF estimations would be minimal in this study. Future attention can be paid to developing new phenotyping systems and using the spectral fitting methods to quantify SIF and SIF yield with higher accuracy. Furthermore, similar to previous remote sensing-based studies, the provision of reflectance and SIF from hyperspectral cameras in this study may suffer biases induced by plant geometry and structure, leaf angle distribution, seasonal characteristics, and background soil. Thus, efforts are still needed to (partly) eliminate these biases to refine the observed relationship between SIF/SIF yield and photosynthesis.

### Conclusion

Accurate quantification of photosynthetic information in a high-throughput manner is of critical importance to harness variation in photosynthetic capacity towards increases in crop yield. Although reflectance spectra (e.g. machine learning+reflectance spectra/spectral indices) have been widely used for quantifying photosynthetic capacity, it remains a question whether SIF-related signals would be a good proxy for photosynthetic capacities. In this study, both SIF and SIF yield were evaluated as a potential indicator for photosynthetic capacity. The results suggested that SIF yield was a better proxy than SIF to estimate photosynthetic capacities *V*_cmax_ and *J*_max_. More specifically, it was observed that on average SIF yield had the ability to explain ~80% variation in *J*_max_ and ~75% variation in *V*_cmax_. The observed negative relationship between SIF yield and photosynthetic capacity was largely attributed to the positive relationship between APAR (i.e. NDVI×PAR) and photosynthetic capacity. The use of SIF yield as a proxy for photosynthetic capacity thus provides an alternative that can supplement existing approaches in estimating photosynthesis at plot level. Future work can be directed to explore internal mechanisms at the leaf/molecular level to disentangle the pathways of photochemistry, NPQ, and SIF that help explain the observed negative relationship between SIF yield and photosynthetic capacity.

## Data Availability

The data supporting the findings of this study are available from the corresponding author, Carl Bernacchi, upon reasonable request.

## Abbreviations

APAR: absorbed photosynthetically active radiation
CC: Pearson’s correlation coefficient
fAPAR: the ratio between APAR and PAR
GPP: gross primary productivity
HTPP: high-throughput phenotyping platform
iFLD: improved Fraunhofer line discrimination
*J*_max_: maximum electron transport rate
NDVI: normalized difference vegetation index
NPQ: non-photochemical quenching
PAR: photosynthetically active radiation
*R*^2^: coefficient of determination
RMSE: root mean square error
SIF: solar-induced fluorescence
*V*_cmax_: maximum carboxylation rate of Rubisco
VNIR: visible and near infra-red
